# Frühe Hilfen im deutschsprachigen Raum – unterschiedliche Wege zum gleichen Ziel

**DOI:** 10.1007/s00103-024-03972-9

**Published:** 2024-11-25

**Authors:** Marion Weigl, Sabine Haas

**Affiliations:** Nationales Zentrum Frühe Hilfen, Gesundheit Österreich GmbH, Stubenring 6, 1010 Wien, Österreich

**Keywords:** Familie, Frühkindliche Entwicklung, Chancengerechtigkeit, Aufsuchende Arbeit, Qualitätssicherung, Family, Early childhood development, Health equity, Outreach work, Quality assurance

## Abstract

Unter „Frühen Hilfen“ wird im deutschsprachigen Raum grundsätzlich ein Gesamtkonzept von Maßnahmen zur Gesundheitsförderung und insbesondere gezielten Frühintervention in Schwangerschaft und früher Kindheit verstanden, das sich vor allem an Familien in belastenden Lebenssituationen richtet. Dieser Artikel bietet einen Überblick über die Frühen Hilfen in Deutschland, Österreich, Schweiz, Liechtenstein und Südtirol.

Während in Deutschland verschiedene Modelle in die Umsetzung gelangt sind und bis heute noch nebeneinander fortbestehen, wurde in Österreich ein einheitliches Modell festgeschrieben. An diesem orientieren sich auch Südtirol und Liechtenstein. Für die Schweiz liegt zwar mittlerweile auch ein Konzept vor, die Umsetzung erfolgt jedoch bislang nur in einzelnen Kantonen. Eine rechtliche Verankerung gibt es bis dato in Deutschland, Österreich und Südtirol. In Deutschland, Österreich und Liechtenstein wurde jeweils ein nationales Zentrum zur laufenden fachlich-wissenschaftlichen Begleitung und Qualitätsentwicklung beauftragt. Auf Ebene der Bundesländer (Deutschland und Österreich) bzw. der Regionen (Südtirol) sind koordinierende Einheiten aktiv, die jedoch teilweise unterschiedliche Aufgaben innehaben. Das Kernelement der freiwilligen, kostenlosen und aufsuchenden Familienbegleitung erfolgt nach dem österreichischen Modell durch ein multiprofessionelles Team, in Deutschland durch definierte Berufsgruppen (Familienhebammen und Familien-Gesundheits- und Kinderkrankenpflegende). Die verfügbaren Daten sowie Ergebnisse aus der Begleitforschung zeigen, dass die Frühen Hilfen ihr Ziel erreichen. Familien werden entlastet, es werden Zugänge zu spezifischen Unterstützungsangeboten erleichtert. Als sozial bzw. sozioökonomisch benachteiligt geltende Familien können großteils gut erreicht werden.

## Abstract (en)

In German-speaking countries, early childhood intervention programmes—known as “Frühe Hilfen”—are generally understood as an overall concept of health promotion measures that particularly target early intervention in pregnancy and early childhood and are primarily aimed at families in stressful life situations. This article provides an overview of early intervention services in Germany, Austria, Switzerland, Liechtenstein and South Tyrol.

While various models have been implemented in Germany and continue to co-exist to this day, a standardised model has been established in Austria. South Tyrol and Liechtenstein also orientate themselves on this model. Although there is now also a concept for Switzerland, it has so far only been implemented in individual cantons. Germany, Austria and South Tyrol have so far enshrined the concept in law. In Germany, Austria and Liechtenstein, a national centre has been commissioned to provide ongoing professional and scientific support and quality development. Coordinating units are active at the level of federal states (Germany and Austria) and regions (South Tyrol), although they have different responsibilities in some cases. The core element of voluntary and free outreach family support is provided by a multi-professional team according to the Austrian model and by defined professional groups in Germany (family midwives and family health and paediatric nurses). The available data and results from the accompanying research show that Frühe Hilfen is achieving its goal. Families are relieved and access to specific support services is facilitated. Families who are considered socially or socio-economically disadvantaged can largely be reached.

## Einleitung

Frühe Hilfen sind eine Angebotsform, die in internationaler Perspektive dem Bereich der „Early Childhood Interventions“ zuzuordnen ist. Frühe Hilfen werden im deutschsprachigen Raum seit Mitte der 2000er-Jahre verstärkt umgesetzt. Am Beginn der systematischeren Entwicklung in Deutschland und Österreich standen einzelne Modell- bzw. Pilotprojekte, die nach schwerwiegenden Fällen von Kindesmisshandlung vorrangig aus Kinderschutzperspektive heraus beauftragt wurden. Das Konzept stieß bald auf breites Interesse und Frühe Hilfen konnten sich als anerkanntes Konzept zur Förderung von Rahmenbedingungen für eine gesunde Entwicklung und ein gewaltfreies Aufwachsen von Kindern etablieren.

In Deutschland wurde 2006 zunächst durch das Bundesministerium für Familie, Senioren, Frauen und Jugend (BMFSFJ) das Aktionsprogramm „Frühe Hilfen“ initiiert, dessen Kern 10 Modellprojekte Frühe Hilfen waren, die in allen 16 Bundesländern erprobt wurden. Dem folgte im Jahr 2012 die gesetzliche Verankerung der Frühen Hilfen im Bundeskinderschutzgesetz, das eine zeitlich befristete Bundesinitiative enthält, die die auf Dauer angelegte Bundesstiftung Frühe Hilfen ab 2018 vorbereitete. Die Finanzierung der Netzwerke Frühe Hilfen und spezifischer Unterstützungsangebote ist durch den „Fonds Frühe Hilfen“ nun dauerhaft gesichert.

Modellprojekte in einzelnen Regionen standen auch am Beginn der Etablierung in Österreich in den Jahren 2009 bis 2014. Darauf folgte – befördert vor allem durch Finanzierungsmöglichkeiten über die Vorsorgemittel der Bundesgesundheitsagentur – eine mehrjährige Phase des Aus- und Aufbaus von regionalen Frühe-Hilfen-Netzwerken in allen Bundesländern. Durch die Bereitstellung von Mitteln aus dem EU-Programm „NextGenerationEU“ gelang es im Herbst 2023 die Flächendeckung des Angebots zu erreichen. Seit 01.01.2024 gibt es auch in Österreich eine gesetzliche Verankerung der Frühen Hilfen, die – zunächst bis Ende 2028 – die Finanzierung eines flächendeckenden und bedarfsgerechten sowie qualitätsgesicherten Angebots der Frühen Hilfen sicherstellt.

Infolge der Umsetzung in Deutschland und Österreich stieg auch in weiteren deutschsprachigen Ländern bzw. Regionen das Interesse an Frühen Hilfen. Südtirol beteiligte sich in Beobachterrolle an österreichischen Grundlagenprojekten zu Frühen Hilfen. Die konkrete Arbeit begann schließlich 2014 mit einem Forschungsprojekt zu den Frühen Hilfen, das 2017 in 2 Pilotprojekte mündete. Die Umsetzung des entwickelten Modells wird seit 2021 auf Basis eines Beschlusses der Landesregierung mit öffentlichen Mitteln in ganz Südtirol ausgebaut, eine Flächendeckung ist mit 2024 angestrebt.

In der Schweiz liegen Forschungsarbeiten zum Thema sowie eine regional begrenzte Umsetzung von Angeboten vor. Eine Vorstudie setzte sich mit den Möglichkeiten der Implementierung von Frühen Hilfen – in der Schweiz unter dem Fachbegriff „Frühe Förderung“ gefasst – auseinander und entwickelte ein Modell der familienzentrierten Vernetzung für die Schweiz.

Liechtenstein beobachtete über längere Zeit die Entwicklungen in Vorarlberg und beteiligte sich 2019 an der 5‑Länder-Tagung zum Thema Frühe Hilfen. In der Folge wurde eine Grundlagenarbeit durchgeführt, die eine klare Empfehlung für ein Programm „Frühe Hilfen“ aussprach [1].

International tut sich im Bereich Early Childhood Interventions ebenfalls sehr viel, wie unterschiedliche Initiativen (siehe unten) und zahlreiche Publikationen zeigen. Ein Gesamtüberblick dazu würde aber den Rahmen des Artikels sprengen, der Fokus liegt daher auf dem deutschsprachigen Raum.

Der folgende Text bietet einen Überblick über die Situation in den verschiedenen deutschsprachigen Ländern/Regionen. Dazu beschreibt er die Gemeinsamkeiten und Unterschiede der vorliegenden bzw. in Umsetzung befindlichen Konzepte und der unterstützenden Strukturen. Dabei wird auch auf die dahinterliegenden Entwicklungsprozesse und ausgewählte Daten bzw. Studienergebnisse eingegangen.

## Gemeinsamkeiten im deutschsprachigen Raum

### Das gemeinsame Ziel

Der Förderung der frühkindlichen Entwicklung wird seit einigen Jahrzehnten große Bedeutung zugesprochen. So werden in einer Bestandsaufnahme zu effektiven Maßnahmen zur Förderung gesundheitlicher Chancengerechtigkeit der Weltgesundheitsorganisation (WHO; [2]) Investitionen in frühkindliche Entwicklung und Bildung sowohl aus gesundheitlicher als auch ökonomischer Perspektive als höchst relevant beschrieben. Betont wird dabei, dass sie die Chance bieten, die Fortschreibung von gesundheitlichen Ungleichheiten von einer Generation auf die nächste zu stoppen und die großen Potenziale der frühen Kindheit für lebenslange Gesundheit wie Lebensqualität zu nutzen.

International ist das Interesse an diesem Präventionsbereich, der im deutschsprachigen Raum unter „Frühe Hilfen“ gefasst ist, stark gestiegen. Unabhängig von der jeweiligen Ausgangslage ist das Ziel in allen Ländern das Herstellen eines familiären und gesellschaftlichen Umfelds, das zur Förderung der frühkindlichen Entwicklung und langfristig zu einem Abbau von gesundheitlicher und sozialer Ungleichheit beitragen soll. Die WHO, das Kinderhilfswerk der Vereinten Nationen (UNICEF) und die Weltbank haben 2018 in Kooperation mit vielen Netzwerken und Partnern dazu das „Nurturing Care Framework^1^“ [3] veröffentlicht. Zusätzlich wurde von der WHO Europa darauf aufbauend eine Leitlinie erarbeitet, in der das Recht der Kinder, ihr volles Entwicklungspotenzial erreichen zu können, betont und – im Einklang mit den UN-Nachhaltigkeitszielen – als grundlegende Voraussetzung für nachhaltige Entwicklung definiert wird.

### Gemeinsame Kernelemente

Im Einklang damit teilen auch die deutschsprachigen Programme der Frühen Hilfen die grundsätzliche *Zielsetzung* der Förderung einer gesunden Entwicklung und der Sicherung eines gewaltfreien Aufwachsens von Kindern sowie von deren Rechten auf Schutz, Förderung und Teilhabe von der frühesten Lebensphase an.

Grundsätzlich wird unter Frühen Hilfen im deutschsprachigen Raum ein Gesamtkonzept von Maßnahmen zur Gesundheitsförderung und gezielten Frühintervention in Schwangerschaft und früher Kindheit, das die Ressourcen und Belastungen von Familien in spezifischen Lebenslagen berücksichtigt, verstanden.

In allen Ländern kommt damit im Bereich der Frühen Hilfen auch der *intersektoralen Kooperation* ein großer Stellenwert zu. In Deutschland und Österreich erfolgte die Etablierung der Frühen Hilfen in sektorenübergreifender Kooperation, allen voran mit Einbindung der Politikbereiche Familie bzw. Kinder- und Jugendhilfe sowie Gesundheit. So ist beispielsweise die sektorenübergreifende Kooperation durch die gemeinsame Trägerschaft des Nationalen Zentrums Frühe Hilfen (NZFH) durch das Deutsche Jugendinstitut und die Bundeszentrale für gesundheitliche Aufklärung auch strukturell verankert. Auch in Südtirol bestand von Anfang an eine sektorenübergreifende Kooperation.

In Hinblick auf die vorrangigen *Zielgruppen* liegt konzeptionell und in der praktischen Umsetzung besonderes Augenmerk auf Familien in belastenden Lebenssituationen und insbesondere auch sozioökonomisch benachteiligten Bevölkerungsgruppen. Diese sollen möglichst frühzeitig erreicht und bedarfsorientiert kurz- bis längerfristig unterstützt werden.

Darüber hinaus gibt es noch weitere Gemeinsamkeiten in der grundsätzlichen *Gestaltung *von Angeboten der Frühen Hilfen: Gleich ist den Programmen im deutschsprachigen Raum die Relevanz von lokalen und regionalen Unterstützungssystemen, die die verfügbaren Hilfsangebote für Eltern und Kinder in der Phase der Schwangerschaft und den ersten Lebensjahren koordinieren, Unterstützung, Beratung und Begleitung bereitstellen und den Zugang zu benötigten weiterführenden Dienstleistungen (inklusive finanzieller Leistungen) erleichtern. Aktivitäten zur Koordination und Vernetzung auf Ebene der Netzwerkpartner ebenso wie eine „Lotsenfunktion“ in der direkten Arbeit mit Familien sind damit integrale Bestandteile der Frühen Hilfen. Außerdem wird niederschwelligen Ansätzen viel Wert zugeschrieben. In allen Programmen wird daher – besonders in Hinblick auf die bedarfsorientierte Unterstützung von sozioökonomisch benachteiligten bzw. generell belasteten Familien – aufsuchende Arbeit (insb. in Form von Hausbesuchen) bereitgestellt.

Nicht zuletzt teilen die Modelle für den deutschsprachigen Raum auch *Grundsätze und Haltungen*. Alle betonen beispielsweise die Bedarfsorientierung und das Ziel der Förderung von Chancengerechtigkeit. In Hinblick auf die Arbeit mit den Familien werden Haltungen wie Ressourcenorientierung, Wertschätzung, Freiwilligkeit, Empowerment sowie die Vermeidung von Stigmatisierung als zentral dargestellt.

### Fachlich-wissenschaftliche Begleitung und Qualitätssicherung

Eine solide konzeptionelle Basis und laufende fachlich-wissenschaftliche Begleitung und Qualitätsentwicklung sind ebenfalls ein geteiltes Anliegen bei den Frühen Hilfen im deutschsprachigen Raum. Grundlagenarbeiten sowie Evaluation und wissenschaftliche Begleitung waren und sind in allen Ländern wichtig.

In Deutschland beschreibt das Leitbild Frühe Hilfen^2^ des NZFH-Beirats aus dem Jahr 2014 anhand von Leitsätzen die wichtigsten Elemente, Charakteristika und Anforderungen an Frühe Hilfen, inklusive Qualitätssicherung und regelmäßiger Evaluation. Dementsprechend wurden die Modellprojekte, die Bundesinitiative und die Bundesstiftung Frühe Hilfen intensiv fachlich und wissenschaftlich begleitet. Das aktuelle Forschungsprogramm umfasst Strukturanalysen [4], Forschung zu Unterstützungsbedarf und Erreichbarkeit von Familien [5], zu sektorenübergreifender Kooperation [6] und zur Wirkung einzelner Interventionen (z. B. [7]).

Fachliche Ausgangsbasis in Österreich war eine in den Jahren 2011 bis 2014 durchgeführte Grundlagenarbeit mit einer sehr umfassenden Feldanalyse, Evidenzanalysen und Stakeholder-Workshops. Auf dieser Grundlage wurde ein „Idealmodell“ Frühe Hilfen [8] entwickelt, das bis heute die zentrale fachliche Basis der Umsetzung darstellt [9]. Seit einigen Jahren steht auch ein Wirkmodell zu Frühen Hilfen zur Verfügung [10]. Externe Evaluationen begleiteten die Umsetzung in allen relevanten Phasen. Darüber hinaus gibt es spezifische Forschungsprojekte und Aktivitäten zur Einbindung der Zielgruppen.

In Südtirol begann die konkrete Befassung mit einer möglichen Umsetzung der Frühen Hilfen auch mit einem Forschungsprojekt, das dann in Pilotregionen resultierte. Es wurde – am österreichischen Konzept orientiert – ein spezifisches Modell für Südtirol erarbeitet [11]. Ausgehend von Empfehlungen einer Grundlagenarbeit wurde auch für Liechtenstein das grundsätzlich als geeignet erachtete österreichische Modell um spezifische Elemente für Liechtenstein ergänzt [1]. Für die Schweiz wurde auf Basis von wissenschaftlicher Befassung ein Basismodell beschrieben, das ebenfalls die stärkere Vernetzung aller Fachleute rund um die Geburt zum Ziel hat [12].

Die laufende bundesweite Begleitung und Qualitätsentwicklung sowie -sicherung erfolgen in Deutschland und Österreich (Gesundheit Österreich GmbH) jeweils durch ein in Bundesinstitutionen angesiedeltes Nationales Zentrum Frühe Hilfen (NZFH bzw. NZFH.at). In Deutschland werden vom NZFH beispielsweise ein Qualitätsrahmen, fachlich fundierte Arbeitshilfen, Kompetenzprofile und Qualifizierungsmodule sowie eine Lernplattform zur Verfügung gestellt.^3^ In Österreich wird die Qualitätssicherung seitens des NZFH.at u. a. durch einen Qualitätsstandard Frühe Hilfen [13], ein Schulungs- und Fortbildungsangebot und die Entwicklung zahlreicher weiterer fachlicher Grundlagen (Leitfäden etc.) gefördert.^4^

Beide NZFH erarbeiten zunehmend auch Informationsmaterialien für Familien, um den Bedarf an leicht verständlichen Informationen, der im Zuge der Umsetzung der Frühen Hilfen immer wieder festgestellt wurde, abzudecken (https://www.elternsein.info/ bzw. https://fruehehilfen.at/).

In Hinblick auf Rolle und Aufgabenumfang vergleichbare Institutionen stehen in den anderen Ländern bzw. Regionen nicht zur Verfügung. Fachliche Begleitung und Qualitätssicherung werden aber auch dort als wichtig erachtet. In Südtirol wurden die fachliche Begleitung und die Koordination in den ersten Jahren vom Forum Prävention übernommen [11], seit 2023 liegt die zentrale Koordinationsfunktion beim Land. In Liechtenstein ist eine Fachstelle neben anderen Aufgaben auch für die Qualitätsentwicklung auf struktureller Ebene zuständig [1].

Insgesamt kann damit festgestellt werden, dass im deutschsprachigen Raum die grundsätzlichen Ziele von Frühen Hilfen geteilt werden. Dies nicht nur bezogen auf die Zielgruppen und damit die beabsichtigte Wirkung des Angebots, sondern auch auf zentrale Elemente der Konzeption entsprechender Interventionen bzw. auch der strategischen Vorgehensweise zur Implementierung.

## Die unterschiedlichen Wege

Bei der Betrachtung der genauen Ausgestaltung und spezifischer Aspekte der Umsetzung der Frühen Hilfen in den einzelnen Ländern bzw. Regionen zeigen sich aber trotzdem relevante Unterschiede. Spezifische Rahmenbedingungen sowohl in Hinblick auf die jeweiligen Anforderungen zu Beginn der Befassung mit Frühen Hilfen als auch die politisch-administrativen Voraussetzungen erfordern eine jeweils angepasste strategische wie operative Vorgehensweise bzw. auch Anpassungen in Hinblick auf die Konzeption der Intervention. Das heißt, es braucht teilweise unterschiedliche Wege, um im jeweiligen Kontext das gleiche Ziel erreichen zu können.

### Der jeweilige politisch-strategische Rahmen

Wie eingangs ausgeführt, wurden die ersten vom Bund geförderten Modellprojekte in Deutschland und das Pilotprojekt in Österreich aus einer Kinderschutzperspektive heraus initiiert. Eine Perspektive des präventiven Kinderschutzes stand in Deutschland auch im weiteren Aus- und Aufbau der Frühen Hilfen mit im Fokus und die Qualitätsentwicklung im Kinderschutz gehört auch zum Aufgabenspektrum des NZFH. In Österreich bildeten hingegen die in sektorenübergreifenden Prozessen erarbeiteten Public-Health-Strategien „Kinder- und Jugendgesundheitsstrategie“ bzw. „Gesundheitsziele Österreich“ die Grundlage für die Entwicklung eines Modells aus Public-Health-Perspektive. Der breitere Auf- und Ausbau des Angebots wurde auch vorrangig durch Finanzierungsquellen aus dem Gesundheitsbereich ermöglicht. Das Nationale Zentrum Frühe Hilfen (NZFH.at) ist von dem für das Gesundheitsressort zuständigen Bundesministerium beauftragt worden.

Die Initiative für Südtirol erfolgte zunächst durch das Forum Prävention, das sowohl Bezüge im Familien- als auch im Gesundheitsbereich hat, und die Umsetzung wurde seitens der Verwaltung von Anfang an als „soziosanitäre“ Kooperation festgelegt. So lag schon die Koordinierung der Modellphase bei der Abteilung Soziales (Amt für Kinder- und Jugendschutz und soziale Inklusion) und dem Südtiroler Sanitätsbetrieb. Hingegen liegt in Liechtenstein die Verantwortung für die Frühen Hilfen auf Verwaltungsebene im Familienbereich. Im Schweizer Modell wird der Kinder- und Erwachsenenschutzbehörde eine zentrale Rolle zugeschrieben, in Hinblick auf die regionale Koordination werden aber regionale Adaptierungen als notwendig erachtet [12].

Grundsätzlich soll nochmals betont werden, dass trotz der Unterschiede bei der vorrangigen Verortung allen Ländern gemein ist, dass die Kooperation über verschiedene Politikfelder hinweg als zentral für die strategische wie operative Umsetzung der Frühen Hilfen erachtet wird.

### Unterschiede in der Gestaltung des Angebots

Wie oben ausgeführt, gibt es einige konzeptionelle Gemeinsamkeiten in Hinblick auf die Umsetzung von Frühen Hilfen. Unter Frühen Hilfen wird grundsätzlich nicht eine spezifische Leistung oder Intervention verstanden, sondern eine Vielfalt an unterstützenden Maßnahmen in der frühen Kindheit, die in Form von lokalen und regionalen Netzwerken organisiert und bedarfsorientiert zur Verfügung gestellt werden.

In Deutschland entstanden auf dieser Basis regional unterschiedliche Modelle, die mit dem Ziel einer guten Passung mit bestehenden Angeboten und Rahmenbedingungen eine gewisse konzeptionelle Vielfalt aufweisen. Um Fördermittel zu erhalten, mussten aufbauend auf bestehendem Angebot ergänzende und sinnvoll den Bedingungen vor Ort angepasste neue Angebote implementiert werden. Auf dieser Basis wurden seit Beginn verschiedene Formen der Unterstützung als Frühe Hilfen gefördert wie Familienhebammen, Müttercafés, Besuchsdienste, Beratungsangebote, Lotsendienste. Da insbesondere belastete Familien durch niederschwellige Angebote gut erreicht werden können, wurde der Ausbau aufsuchender Angebote wie Familienhebammen und Familien-Gesundheits- und Kinderkrankenpfleger:innen verstärkt gefördert. Die Organisation all dieser Angebote in Form von kommunalen Netzwerken ist ein zentrales Element, um fachlichen Austausch und Zusammenarbeit zu sichern und die Basis für die Entwicklung einer bedarfsgerechten Angebotsstruktur zu legen [14].

Das österreichische „Idealmodell“ [9] gibt eine einheitliche Rahmenkonzeption für „regionale Frühe-Hilfen-Netzwerke“ vor. Unter Frühen Hilfen werden nur Angebote gefasst, die die darin definierten Kernfunktionen erfüllen. In Hinblick auf die Arbeit mit den Familien beinhaltet dies eine aufsuchende „Familienbegleitung“, die insbesondere Familien in belastenden Lebenssituationen zur Seite steht. Die Funktion integriert die Aufgabe, eine Beziehung herzustellen und Vertrauen aufzubauen, mit einer Lotsenfunktion. In Österreich sind die Familienbegleiter:innen Teil eines multiprofessionellen Teams, können damit verschiedenste berufliche Hintergründe (mit Qualifizierung in einem Gesundheits‑, Sozial- oder pädagogischen Beruf) haben, sind jedoch alle bei einem (meist freien, teilweise auch öffentlichen) Träger angestellt.

Wie ausgeführt, orientieren sich Südtirol und Liechtenstein in der Umsetzung stark am österreichischen Konzept. Die regionalen Teams in Südtirol decken ebenfalls verschiedene Berufsgruppen ab, wobei jedenfalls die Bereiche Gesundheit und Soziales vertreten sind [15]. Als ein ergänzendes „Schlüsselelement“ des einheitlichen liechtensteinischen Modells wird ein Screening durch Hebammen, Elternberater:innen und Ärztinnen/Ärzte zur Einschätzung der Belastungssituation gesehen [16].

Die Schweizer Vorstudie empfiehlt auch ein Basismodell, es werden aber – aufgrund der Notwendigkeit, aus bestehenden Strukturen aufzubauen und regionale Unterschiede zu berücksichtigten – 5 Modellvarianten abgeleitet, die lokal adaptiert werden können [12].

In allen Ländern und Regionen sind Koordination und Vernetzung ein zentraler Aspekt der Frühen Hilfen. Aber hinsichtlich der konkreten Gestaltung dieser Aufgaben zeigen sich ebenfalls Unterschiede. In Österreich ist das Netzwerkmanagement integraler Bestandteil des Konzepts. Dies schließt Aktivitäten zum Netzwerkaufbau, zur Sensibilisierung und zur laufenden Netzwerkpflege mit ein. Hingegen wird die Aufgabe des Netzwerkmanagements, inklusive der Netzwerkarbeit, in Südtirol vorrangig vom gesamten regionalen Team übernommen. In Deutschland wird die Verantwortung für das Netzwerkmanagement und damit für Aufgaben in Hinblick auf Vernetzung und Kooperation von den Koordinierungsstellen wahrgenommen, die großteils beim örtlichen Jugendamt angesiedelt sind [17]. In Liechtenstein ist eine Stelle zugleich für das Case-Management als auch das Netzwerkmanagement zuständig.

### Unterschiede in Steuerung und bei begleitenden Strukturen

Im Einklang mit spezifischen Rahmenbedingungen unterscheiden sich auch die begleitenden Strukturen. In Deutschland gibt es eine Geschäftsstelle der Bundesstiftung Frühe Hilfen, die zur Weiterentwicklung der Frühen Hilfen eng mit den Bundesländern zusammenarbeitet. Das NZFH unterstützt die Bundesstiftung Frühe Hilfen durch Maßnahmen zur Qualitätssicherung, Qualitätsentwicklung, zur Begleitforschung und zum Wissenstransfer. Die Bundesstiftung wird von einem Beirat beraten.^5^ Die kommunalen Umsetzer:innen werden von Netzwerkkoordinierenden und Landeskoordinierungsstellen der Bundesländer unterstützt.^6^ Überregionale Netzwerkkonferenzen dienen dem Austausch zwischen den regionalen Umsetzer:innen im ganzen Land. Die auf regionaler Ebene etablierten Koordinierungsstellen sind u. a. auch für Gremienbetreuung und Koordination des Einsatzes von Gesundheitsfachkräften zuständig.

Die Steuerung auf nationaler Ebene wie Bundeslandebene erfolgt in Österreich durch intersektoral besetzte Koordinierungsgruppen. In jedem Bundesland gibt es eine Frühe-Hilfen-Koordination. Das NZFH.at hat ähnliche Aufgaben wie das NZFH und ist zusätzlich noch für Dokumentation und Monitoring sowie Schulung und Fortbildung zuständig. Ein auf nationaler Ebene angesiedelter Fachbeirat berät das NZFH.at in fachlichen Fragen, auf regionaler Ebene werden dazu Expert:innengremien empfohlen. Der Austausch zwischen den regionalen Umsetzer:innen wird vom NZFH.at im Rahmen von Vernetzungstreffen gefördert.

In Südtirol wurden auf Landesebene eine intersektoral besetzte Steuerungsgruppe und ein Frühe-Hilfen-Rat etabliert. Die regionalen Frühe-Hilfen-Teams werden von Frühe-Hilfen-Arbeitsgruppen unterstützt [11], die aus regionalen Stakeholdern bestehen. Die Koordination wird meist von 2 Personen, je eine aus den Bereichen Gesundheit und Soziales, wahrgenommen und stellt die Verbindung zwischen dem Frühe-Hilfen-Team und der Arbeitsgruppe dar.

Die Umsetzung der Frühen Hilfen in Liechtenstein erfolgt durch 2 unterschiedliche Trägerorganisationen. Bei Netzwerk Familie Liechtenstein ist eine Fachstelle u. a. für Vernetzung und Qualitätsentwicklung auf struktureller Ebene zuständig.

Für die Schweiz wird in der Vorstudie eine neue Koordinationsstelle auf nationaler Ebene empfohlen, die die Entwicklung von lokalen Modellen unterstützen und von verschiedenen Interessensverbänden, Wissenschaft und Praxis gemeinsam umgesetzt werden sollte. Eine nationale Strategie zur Verankerung von „Frühen Hilfen“ wird für die Schweiz aber aufgrund der föderalistischen Strukturen als kaum realisierbar erachtet [12].

## Erfolgreiche Erreichung der Ziele trotz unterschiedlicher Wege

Die verfügbaren Daten zeigen, dass es in allen deutschsprachigen Ländern und Regionen gelungen ist, das Angebot der Frühen Hilfen erfolgreich zu etablieren und Familien mit Bedarf zu erreichen und zu unterstützen.

Für *Deutschland* lässt sich dies aus Forschungsarbeiten ableiten. Laut Erhebungen sind Netzwerke Frühe Hilfen inzwischen in fast jeder Kommune mit einem Jugendamt in Deutschland vorhanden und auch die längerfristig aufsuchende Betreuung und Begleitung von Familien durch Gesundheitsfachkräfte als ein Kernangebot der Frühen Hilfen gibt es inzwischen in 97 % der Kommunen [4]. Auch der Anteil von größeren Geburtskliniken, die über einen Lotsendienst verfügen, steigt stetig [18]. Daten der bundesweit repräsentativen Befragung von Familien mit Kindern bis zum Alter von 3 Jahren „Kinder in Deutschland – KiD 0–3“ belegen zudem, dass ein relevanter Anteil von Familien die Frühen Hilfen nutzt. Das gilt auch für die wichtige Zielgruppe von sozioökonomisch benachteiligten Familien. So nahmen 2022 Familien, die in Armut lebten, mit 14,5 % häufiger eine längerfristig aufsuchende Betreuung und Begleitung durch eine Gesundheitsfachkraft in Anspruch als Familien ohne Armutsbelastung (9,5 %) und bewerten sie als hilfreich [5].

In *Österreich* gelang es, in den letzten Jahren ein flächendeckendes Angebot aufzubauen. Nähere Einblicke in die unterstützten Familien erlaubt das österreichweite Dokumentationssystem FRÜDOK. Im Jahr 2023 wurden insgesamt 3674 Familien längerfristig begleitet und weitere 709 Familien kurzfristig unterstützt. Die Anzahl der Neubegleitungen lag bei 2336 Familien, was rund 2,8 % aller Geburten entspricht und damit weiterhin unter dem angestrebten Prozentsatz von ca. 7 % aller Geburten liegt. Über die Jahre begannen jeweils etwa 20–25 % der Familienbegleitungen bereits in der Schwangerschaft, sozioökonomisch benachteiligte Familien werden im Vergleich mit der Gesamtbevölkerung überdurchschnittlich gut erreicht. Das Feedback der begleiteten Familien belegt sehr große Zufriedenheit mit der Familienbegleitung [19]. Evaluationen zeigen sowohl eine Steigerung der verfügbaren Ressourcen (wie soziales Netzwerk, Selbstwertgefühl, Familienklima, Erziehungskompetenz) als auch eine Verringerung der Belastungen (z. B. Zukunftsängste, finanzielle Notlage, psychosoziale Belastungen, soziale Isolation) der begleiteten Familien. Vor allem infolgedessen verbessert sich auch die Eltern-Kind-Bindung und -Interaktion und wird die Entwicklung des Kindes gefördert [20].

Die Evaluationsergebnisse zu 2 Pilotsprengeln^7^ zeigen für *Südtirol* [15], dass es relativ rasch gelungen ist, in Kontakt mit Familien zu treten und diese zu begleiten. 45 % der Kinder waren noch in ihrem ersten Lebensjahr. Die Anliegen der Familien waren vielfältig: insbesondere Überforderung, (psychische) Belastungen, Isolation und Regulationsstörungen. Die Evaluation bestätigte den Bedarf an niederschwelligen und an die Lebenswelt der Familien angepassten Unterstützungsangeboten, insbesondere für Familien in belastenden Lebenssituationen. Aber auch die Notwendigkeit an alltagspraktischer Unterstützung wurde aus der Evaluation abgeleitet.

Zur Umsetzung der Frühen Hilfen in *Liechtenstein* liegen Jahresberichte von Netzwerk Familie Liechtenstein vor [21]. Im Jahr 2023 wurden 91 Familien unterstützt (im Vergleich zur Schätzung von 103–129 Schwangeren und Jungfamilien in mehrfach belasteter Lebenssituation pro Jahr [1]), wobei Hausbesuche einen eher kleinen Anteil der Leistungen ausmachten (mehr Telefon, E‑Mail oder Messengerdienste). Die Familien werden auch in Liechtenstein früh erreicht: Bei 54 % der begleiteten Familien war das jüngste Kind bei Begleitungsbeginn unter einem Jahr alt. Psychische Erkrankungen (41 % der Familien) und Einelternhaushalte (21 %) spielen eine große Rolle. Darüber hinaus wird auch in Liechtenstein eine Vielfalt an Belastungen dokumentiert, die von finanziellen über soziale, familiäre Belastungen bis zu körperlichen Belastungen der Eltern oder erhöhten Fürsorgeanforderungen des Kindes reichen.

Für die Schweiz liegen noch keine Daten vor, da bislang nur einzelne Kantone erste Schritte in Richtung Umsetzung gegangen sind.

## Fazit

Die Frühen Hilfen konnten sich im deutschsprachigen Raum gut als durch die öffentliche Hand finanzierter Angebotsbereich etablieren. Die Programme finden auch international Anerkennung: So diente das deutsche Programm als Case Study [22] der internationalen Allianz „Partnership for Maternal, Newborn, and Child Health“ (PMNCH) und das österreichische Programm wurde auf EU-Ebene als Best Practice anerkannt. Begleitforschung zu den Frühen Hilfen aus dem deutschsprachigen Raum bestätigt den Bedarf und den Nutzen dieses Unterstützungsangebots. Die den Frühen Hilfen zugrunde liegenden Annahmen und die damit verbundenen Ziele sind im deutschsprachigen Raum mehr oder weniger identisch. Unterschiede gab bzw. gibt es in der Entwicklung und Gestaltung der Programme. Intersektoral sind sie in jedem Land gedacht und aufgesetzt, wenn auch die Hauptverantwortung entsprechend ursprünglicher Initiative bzw. Rahmenbedingungen unterschiedlich sein kann.

Die verfügbaren Daten und Begleitstudien zeigen, dass die Umsetzung der Frühen Hilfen gelingt. Sie erreichen Familien mit verschiedensten Belastungen, werden von diesen gerne genutzt und können zu ihrer Entlastung beitragen. Finanzielle Problemlagen bzw. Armutsgefährdung sind ein wichtiges Thema, die Frühen Hilfen können den Zugang zu finanzieller Unterstützung erleichtern. Auch psychische Belastungen sind weitverbreitet und können durch Entlastungsgespräche sowie die Vermittlung von weiterführender spezifischer Unterstützung adressiert werden. Damit kann letztendlich ein wichtiger Beitrag zur gesunden Entwicklung der Kinder und langfristig zu gesundheitlicher und sozialer Chancengerechtigkeit geleistet werden.

Die Fachkräfte der Frühen Hilfen (Familienhebammen, Familienbegleiter:innen etc.) genießen aufgrund ihrer Arbeitsweise hohes Vertrauen bei den begleiteten Familien. Sie können daher auch gute und vertiefende Einblicke in die Lebensrealität der Familien gewinnen. Dadurch können sie auf nicht abgedeckte oder schwer abdeckbare Bedarfe hinweisen. Abgesehen davon sehen sie aber auch frühzeitig Folgen gesellschaftlicher Entwicklungen auf Familien und können diese in den gesellschaftlichen bzw. politischen Dialog einbringen.

## Footnotes

1 Ein Rahmenkonzept, das die gesunde Entwicklung von Kindern fördern soll und aus 5 Komponenten besteht: gute Gesundheit, adäquate Ernährung, responsive Betreuung, Sicherheit und Schutz, Gelegenheiten, frühzeitig zu lernen.

2 Siehe https://www.fruehehilfen.de/grundlagen-und-fachthemen/grundlagen-der-fruehen-hilfen/fruehe-hilfen-leitbild-mit-leitsaetzen/ (Zugriff am 01.07.2024).

3 Siehe https://www.fruehehilfen.de/qualitaetsentwicklung-fruehe-hilfen/ (Zugriff am 24.05.2024).

4 Siehe https://nzfh.at/ (Zugriff am 24.05.2024).

5 https://www.fruehehilfen.de/das-nzfh/ziele-und-aufgaben/aufgaben-des-nzfh/ (Zugriff am 08.05.2024).

6 https://www.fruehehilfen.de/grundlagen-und-fachthemen/landeskoordinierungsstellen/ (Zugriff am 08.05.2024).

7 Damit werden in Südtirol kleinere Einheiten als Bezirke bezeichnet, z. B. Brixen und Umgebung.
